# The association between Metabolic Score for Visceral Fat and depression in overweight or obese individuals: evidence from NHANES

**DOI:** 10.3389/fendo.2024.1482003

**Published:** 2024-09-26

**Authors:** Heng Liu, Huqiang Dong, Yu Zhou, Mingchu Jin, Haidong Hao, Yutang Yuan, Hongtao Jia

**Affiliations:** ^1^ Department of Urology, Renmin Hospital, Hubei University of Medicine, Shiyan, Hubei, China; ^2^ School of Public Health, Ningxia Medical University, Yinchuan, Ningxia, China

**Keywords:** Metabolic Score for Visceral Fat, depression, obesity, METS-VF, NHANES

## Abstract

**Background:**

Depression is a common mental illness with a high prevalence in overweight or obese individuals. Recent studies suggest that the Metabolic Score for Visceral Fat (METS-VF) is a novel metric for assessing visceral fat levels, potentially linking metabolic disturbances to depression. This study explores the association between METS-VF and depression severity in overweight or obese U.S. adults.

**Methods:**

Data were obtained from the National Health and Nutrition Examination Survey (NHANES) 2007-2018 dataset, including 9,415 overweight or obese participants. Depression severity was measured using the Patient Health Questionnaire-9 (PHQ-9). To assess the association between METS-VF and depression, the study methodology included multivariate logistic regression, subgroup analyses, generalized additive model (GAM), and smoothed curve fitting. This study also calculated BMI for the Non-Hispanic Asian population from 2011-2018 and incorporated this data as part of a sensitivity analysis.

**Results:**

Elevated levels of METS-VF in overweight or obese participants were significantly associated with increased PHQ-9 scores and an increased likelihood of depression. Notably, this association remained significant after adjustment for multiple covariates. Smoothed curve-fitting plots showed no nonlinear association between METS-VF and PHQ-9 scores. Subgroup analyses confirmed the robustness of these results across populations, particularly among people under the age of fifty. The sensitivity analyses confirmed the robustness of the results in this study.

**Conclusion:**

METS-VF levels were positively associated with depression severity and the likelihood of depression in overweight or obese individuals, with the association being particularly pronounced in people under 50 years of age.

## 1 Background

Depression is a prevalent mental illness marked by persistently low mood, sleep or eating disturbances, excessive fatigue, loss of interest in activities, and intense feelings of guilt (1, 2). At now, estimates put the number of individuals affected by the illness at around 300 million globally, and the prevalence is rising annually (3). In terms of death, depression is just second to heart disease (4). Depression imposes a severe economic burden on individuals, families, society, and the healthcare system. It also has a serious impact on patients’ quality of life and leads to an increased risk of suicide (2, 5). However, studies have shown that the treatment of depression often lacks effectiveness and has a high rate of recurrence (6). Therefore, it is necessary to explore the influencing factors that may contribute to the development of depression and to provide early intervention.

Psychological, social, and physiological variables are among the risk factors for depression. Research has demonstrated a high correlation between obesity and the onset of depression among physiological parameters (7, 8). When it comes to depression in women, those who are obese are more likely to experience it than those who are not (1, 9). It has been shown that obesity significantly impacts diseases such as cardiovascular disease and cancer (10–12). It is also associated with psychiatric conditions, including mental and mood disorders (13, 14). These conditions, in turn, can lead to unhealthy diets and exacerbate the occurrence of overweight or obesity (15). BMI is a commonly used indicator of obesity. Nevertheless, its use in evaluating the body’s distribution of adipose tissue is limited (16). Therefore, a new index of obesity has been developed, called the Metabolic Score of Visceral Fat (METS-VF) (17, 18). This index has been shown to significantly measure other diseases, including fatty liver and diabetes, compared to other measures of obesity (19, 20). The connection between METS-VF and depression is still not well understood, despite significant advancements. By exploring the intricate connections between metabolic health and mental well-being, we can unlock new pathways for early intervention and tailored treatment approaches.

## 2 Materials and methods

### 2.1 Study population

**Figure 1:**
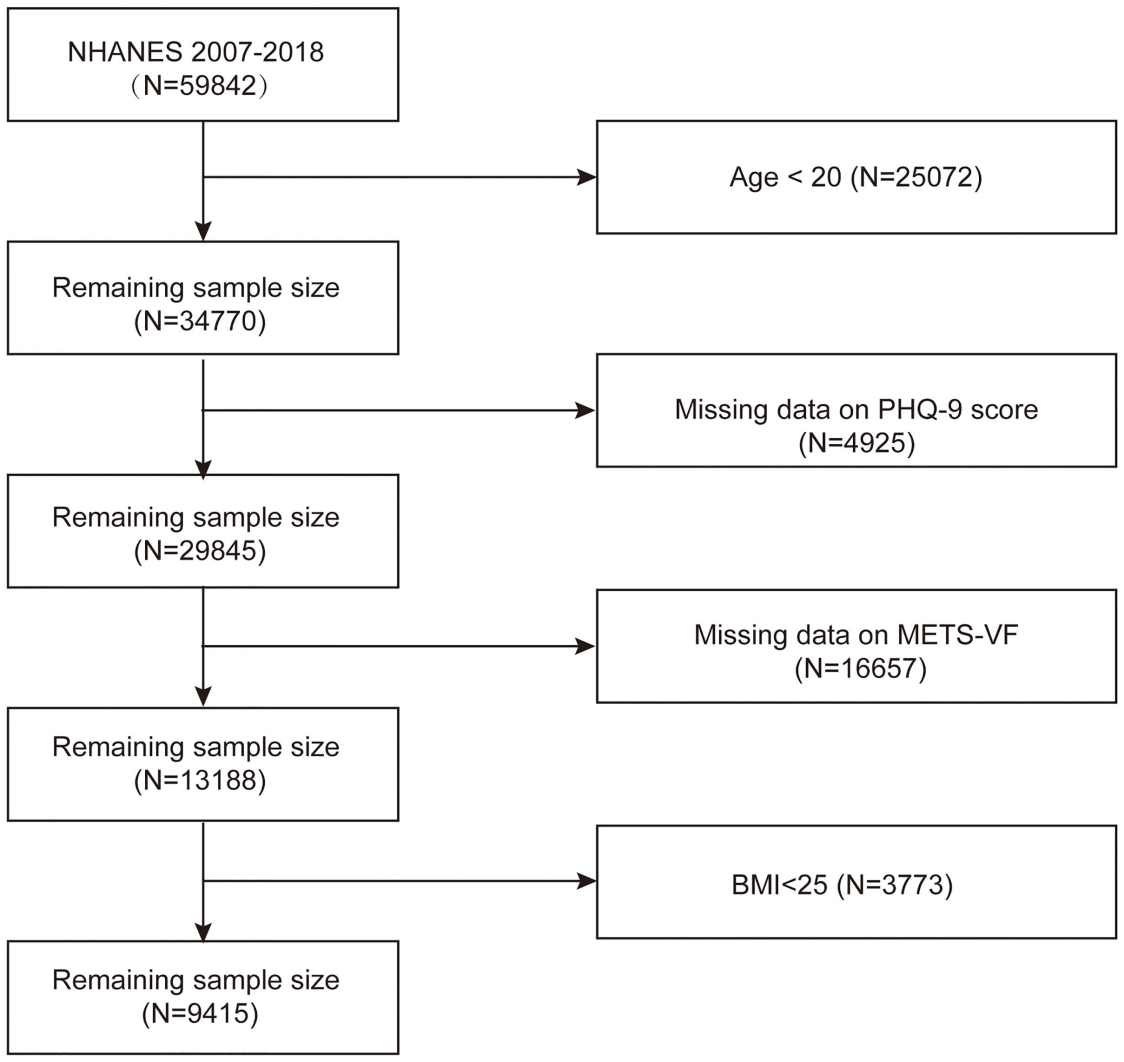
Flow chart of sample selection from the NHANES 2007–2018.

NHANES, a project by the National Center for Health Statistics (NCHS), continuously assesses the health and nutritional status of the U.S. population. Trained personnel collected comprehensive data on socio-economic status, demographics, diet, and health from NHANES participants. In the present study, we downloaded six consecutive datasets (2007-2008, 2009-2010, 2011-2012, 2013-2014, 2015-2016, and 2017-2018) from the website to precisely evaluate the association between METS-VF and depression in overweight or obese patients. Of the total sample of 59,842 participants, 50,427 were excluded due to missing PHQ-9 scores and METS-VF data, having a BMI < 25, or being under 20 years old. Finally, 9,415 US patients who were overweight or obese were included in the analyses (**Figure 1**).

### 2.2 Assessment of depression

The PHQ-9 is a widely used depression screening questionnaire with 9 items, each scored from 0 to 3, resulting in a total score between 0 and 27 (21). A score of 10 or more suggests the presence of depression and is a validated threshold commonly used in epidemiological studies (22).

### 2.3 Evaluation of the METS-VF

The METS-VF assesses exposure to adipose tissue, incorporating the insulin resistance index (METS-IR), waist-to-height ratio (WHTR), age, and sex. At the Mobile Examination Center, professional technicians measure BMI, waist circumference, and height. HDL-C and triglycerides are analyzed with the Cobas 6000 Chemistry Analyzer, and fasting blood glucose is measured using the Roche/Hitachi Cobas C311 Chemistry Analyzer. Measurements use specific units: FBG and HDL-C in mg/dL, TG in mg/dL, BMI in kg/m², age in years, and sex coded as male = 1, female = 0. The METS-VF was calculated using the following formula (18):


$$WHTR=\frac{WC}{Height}$$

$$METS-IR=\frac{ln\left(\left(2\times FBG+TG\right)\times BMI\right)}{ln\left(HDL-C\right)}$$

$$\begin{array}{c} \text{METS}-VF=4.466+0.01\times {\left(\ln(\text{METS}-\text{IR })\right)}^{3}+3.329\times {\left(\ln(\text{WHTR})\right)}^{3}\\ \;\;\;\;+0.319\times \text{gender}+0.594\times \ln(\text{age}) \end{array}$$

### 2.4 Assessment of overweight and obesity

According to the recommendations of the World Health Organization (WHO), overweight and obese adults are classified based on BMI, which is defined as weight divided by height squared (kg/m²). The classifications are as follows: overweight BMI: 25-29.9 kg/m²; obese BMI ≥30 kg/m². However, Asian Americans face a higher risk of metabolic disease than other populations at the same BMI level due to a tendency toward central obesity. For this population, the classifications are adjusted as follows: overweight BMI: 23-27.5 kg/m²; obese BMI: ≥27.5 kg/m² (23–25).

As detailed data on Non-Hispanic Asian populations in the NHANES database only became available after 2010, we extracted NHANES data from 2011-2018 to re-analyze the association between METS-VF and depression in overweight or obese populations in order to avoid substantial missing sample sizes as part of a sensitivity analysis.

### 2.5 Covariates

This study included covariates such as gender, age, race, education level, marital status, household poverty-to-income ratio, BMI, smoking status, alcohol use, diabetes, hypertension, CVD, and stroke. Smokers were defined as those who had smoked at least 100 cigarettes in their lifetime and were smoking during the survey. Alcohol drinkers have consumed at least 12 alcoholic beverages in their lifetime and any year. Diabetes mellitus was identified by a physician’s diagnosis or fasting blood glucose ≥126 mg/dL. Hypertension was noted for individuals with a prior diagnosis, on medication, or with systolic/diastolic blood pressure ≥140/90 mmHg. CVD included those diagnosed with congestive heart failure, coronary artery disease, angina pectoris, or heart attack.

### 2.6 Statistical analyses

The NHANES multi-stage design was accounted for in all statistical analyses. Continuous variables were presented as survey-weighted means with 95% confidence intervals (CI), while categorical variables were shown as survey-weighted percentages with 95% CI. Differences between depressed and non-depressed groups were assessed using weighted linear regression or weighted chi-square tests.

To explore the association between METS-VF and depression in overweight or obese patients, three logistic regression models were used: Model 1 was unadjusted. Model 2 was adjusted for age, sex, and race. Model 3 was further adjusted for education, marital status, BMI, PIR, smoking, alcohol use, diabetes, hypertension, CVD, and stroke. Weighted multiple regression analyses were conducted to describe the association between METS-VF and PHQ-9 depression scores, treating METS-VF as both continuous and categorical (quartiles) variables and estimating trends by treating quartiles as continuous variables. The non-linear relationship between METS-VF and depression prevalence was explored using a generalized additive model (GAM) and smooth curve fitting. When non-linear associations were found, two-segment linear regression models were compared to single-linear models using a log-likelihood ratio test, and threshold effects were calculated. Subsequently, subgroup analyses and interaction tests were performed on potential confounders listed in the baseline table. The study was statistically analyzed using R version 4.3.3 and Empower software with the significance level set at *P* < 0.05.

## 3 Results

### 3.1 Baseline characteristics of the study population

The weighted prevalence of depressed patients (PHQ-9 score ≥10) was 7.82% (7.09%, 8.63%). Compared with non-depressed patients, depressed patients had higher METS-VF levels of 6.55 (6.52, 6.57), *P* < 0.01. The depressed group had a higher prevalence of female patients, individuals who were divorced or living alone, and those with cardiovascular disease, diabetes mellitus, stroke, and hypertension. Conversely, smoking, alcohol consumption, educational attainment, and PIR levels were significantly lower in the depressed group, with a notable difference between the groups (*P* < 0.01) (**Table 1**).

**Table 1:** Weighted characteristics of the study population based on depression.

Characteristics	Total (n = 9415)	PHQ-9 <10 (n = 8538)	PHQ-9 ≥10 (n = 877)	*P*-value
Age (years)	49.08 (48.56,49.59)	49.10 (48.54,49.65)	48.86 (47.64,50.09)	0.738
Gender (%)				<0.0001
Male	51.52 (50.41,52.63)	53.10 (51.95,54.25)	32.94 (29.02,37.10)	
Female	48.48 (47.37,49.59)	46.90 (45.75,48.05)	67.06 (62.90,70.98)	
Race (%)				0.001
Mexican American	9.94 (8.27,11.92)	10.02 (8.33,12.00)	9.09 (6.83,12.01)	
Other Hispanic	6.24 (5.19,7.49)	5.99 (4.97,7.20)	9.25 (6.74,12.58)	
Non-Hispanic White	67.26 (64.17,70.20)	67.76 (64.65,70.71)	61.38 (55.52,66.93)	
Non-Hispanic Black	10.89 (9.39,12.59)	10.65 (9.15,12.36)	13.65 (11.17,16.57)	
Other Race	5.67 (4.91,6.54)	5.59 (4.83,6.46)	6.62 (4.53,9.58)	
Education level (%)				<0.0001
Less than high school	16.46 (15.16,17.85)	15.62 (14.31,17.04)	26.34 (23.04,29.93)	
High school	23.87 (22.45,25.36)	23.58 (22.12,25.10)	27.37 (23.69,31.39)	
More than high school	59.67 (57.55,61.74)	60.80 (58.61,62.95)	46.29 (41.92,50.72)	
Marital status (%)				<0.0001
Never married	15.06 (13.77,16.45)	14.83 (13.47,16.30)	17.79 (14.53,21.59)	
Married/Living with partner	65.88 (64.19,67.54)	67.33 (65.52,69.09)	48.85 (43.77,53.95)	
Widowed/divorced/Separated	19.05 (17.98,20.18)	17.84 (16.72,19.02)	33.36 (29.36,37.62)	
PIR (%)				<0.0001
<1.3	22.39 (20.84,24.02)	20.49 (19.07,21.99)	44.80 (39.66,50.05)	
1.3 - 3.5	36.09 (34.48,37.74)	36.22 (34.56,37.91)	34.59 (30.50,38.91)	
≥3.5	41.51 (39.27,43.80)	43.29 (41.04,45.57)	20.62 (16.42,25.56)	
BMI (%)				<0.0001
25 - 30	47.23 (45.97,48.50)	48.35 (47.00,49.71)	34.02 (30.09,38.19)	
≥30	52.77 (51.50,54.03)	51.65 (50.29,53.00)	65.98 (61.81,69.91)	
Smoking status (%)				<0.0001
Never	54.55 (53.01,56.08)	55.78 (54.13,57.41)	40.08 (36.18,44.11)	
Now	17.18 (16.10,18.32)	15.66 (14.59,16.79)	35.12 (30.44,40.10)	
Former	28.27 (26.75,29.84)	28.57 (27.03,30.15)	24.80 (20.63,29.51)	
Alcohol intake (%)				0.0016
No	10.11 (8.90,11.47)	9.73 (8.52,11.08)	14.64 (11.36,18.67)	
Yes	89.89 (88.53,91.10)	90.27 (88.92,91.48)	85.36 (81.33,88.64)	
Hypertension (%)				<0.0001
No	55.04 (53.50,56.58)	55.87 (54.24,57.50)	45.27 (40.89,49.72)	
Yes	44.96 (43.42,46.50)	44.13 (42.50,45.76)	54.73 (50.28,59.11)	
Diabetes (%)				<0.0001
No	81.19 (80.08,82.24)	81.78 (80.57,82.93)	74.22 (70.92,77.27)	
Yes	18.81 (17.76,19.92)	18.22 (17.07,19.43)	25.78 (22.73,29.08)	
Stroke (%)				<0.0001
No	97.03 (96.59,97.42)	97.40 (96.89,97.82)	92.75 (90.18,94.68)	
Yes	2.97 (2.58,3.41)	2.60 (2.18,3.11)	7.25 (5.32,9.82)	
CVD (%)				<0.0001
No	92.00 (91.29,92.67)	92.52 (91.75,93.23)	85.94 (82.98,88.46)	
Yes	8.00 (7.33,8.71)	7.48 (6.77,8.25)	14.06 (11.54,17.02)	
FBG (mg/dl)	110.57 (109.61,111.52)	110.04 (109.06,111.03)	116.72 (113.43,120.01)	0.0003
HDL-C (mg/dl)	50.95 (50.45,51.44)	51.06 (50.55,51.57)	49.63 (48.56,50.71)	0.011
TG (mg/dl)	137.99 (134.48,141.50)	136.53 (133.02,140.04)	155.20 (145.51,164.89)	0.0002
WHTR	0.63 (0.63,0.63)	0.63 (0.63,0.63)	0.67 (0.66,0.67)	<0.0001
METS-IR	2.40 (2.39,2.41)	2.40 (2.39,2.40)	2.45 (2.43,2.47)	<0.0001
METS-VF	6.50 (6.49,6.51)	6.50 (6.48,6.51)	6.55 (6.52,6.57)	0.0033

For continuous variables: survey-weighted mean (95% CI), with the P-value determined by survey-weighted linear regression. For categorical variables: survey-weighted percentage (95% CI), with the P-value determined by survey-weighted Chi-square test.

PIR, the ratio of income to poverty; BMI, body mass index; HDL-C, high-density lipoprotein cholesterol; TG, triglyceride; WHTR, waist-to-height ratio; FBG, fasting blood glucose; CVD, cardiovascular disease; METS-IR, metabolic score for insulin resistance; METS-VF, metabolic score for visceral fat; PHQ-9, Patient Health Questionnaire-9.

### 3.2 Association between METS-VF and depression

**Figure 2:**
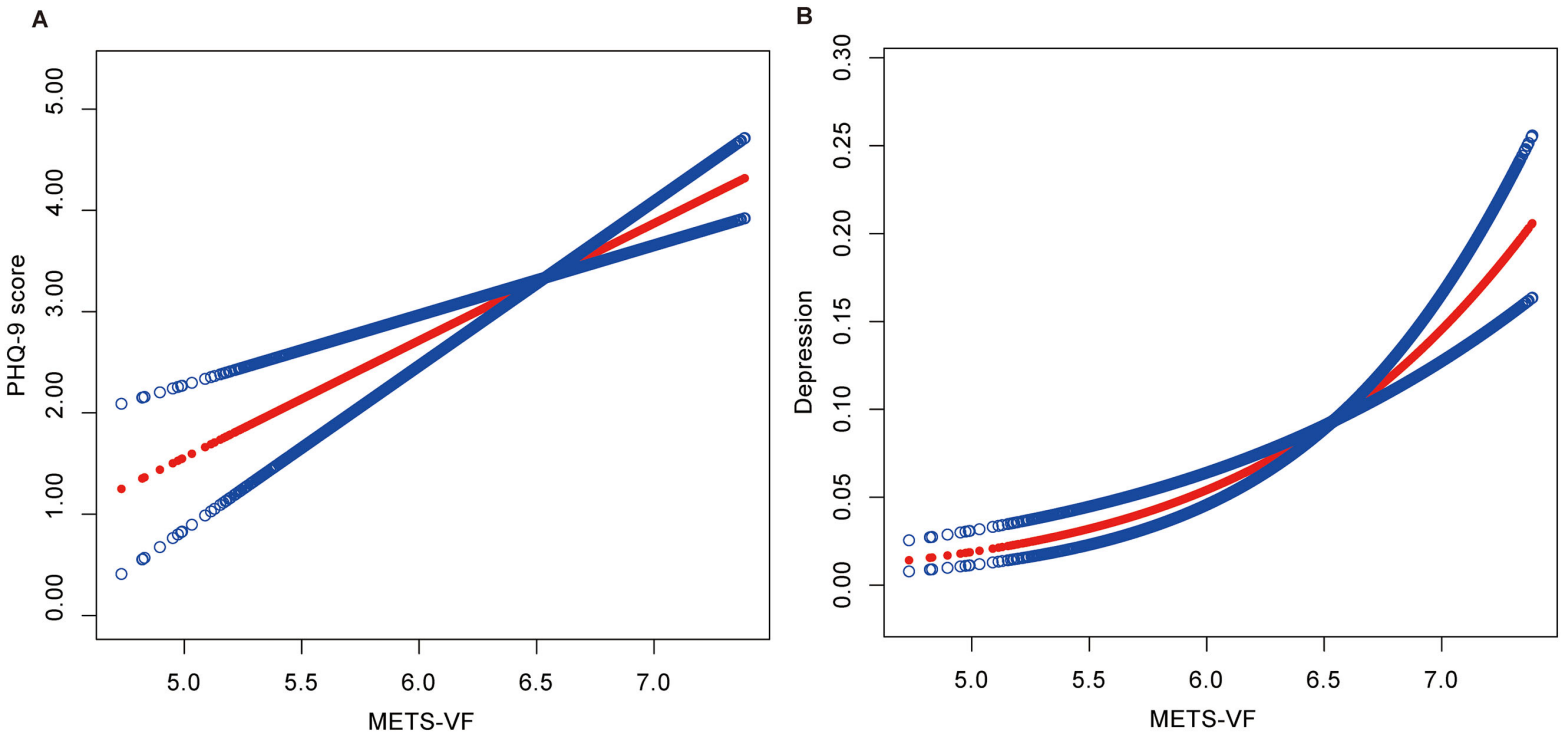
**(A)** Smooth curve fitting between METS-VF and PHQ-9 Score. **(B)** Smooth curve fitting between METS-VF and Depression. The associations were adjusted for gender, age, race, education level, marital status, PIR, smoking status, alcohol drinking status, diabetes status, hypertension status, CVD, and stroke.

**Table 2** shows a positive correlation between METS-VF and PHQ-9 scores, with (β=0.28, 95% CI: 0.06, 0.51) in the crude model and (β=1.45, 95% CI: 1.11, 1.79) in the fully adjusted model. Similarly, METS-VF was positively associated with depression, where a one-unit increase in METS-VF raised the likelihood of developing depression by 197% (OR=2.97, 95% CI: 2.13, 4.14) after adjusting for all covariates. Additionally, when METS-VF was divided into quartiles, we observed that the positive association persisted and became more pronounced with higher METS-VF levels (*P* for trend <0.05). Smoothed curve-fitting analyses indicated no non-linear relationship between METS-VF and depression or PHQ-9 scores (log-likelihood ratio test *P*-value > 0.05) (**Figure 2**) (**Table 3**).

**Table 2:** Multiple regression analysis between METS-VF and depression in overweight or obese patients.

METS-VF	PHQ-9 score	Depression
	β(95%CI)	OR (95%CI)
Crude model (model 1)		
Continuous	0.28 (0.06, 0.51)	1.39 (1.16, 1.66)
Categories		
Quartile1	0(ref)	1(ref)
Quartile2	0.45 (0.20, 0.70)	1.37 (1.12, 1.69)
Quartile3	0.53 (0.28, 0.78)	1.58 (1.29, 1.94)
Quartile4	0.18 (-0.07, 0.43)	1.27 (1.03, 1.56)
*P* for trend	0.120	0.013
Minimally adjusted model (model 2)		
Continuous	2.17 (1.84, 2.51)	4.86 (3.55, 6.66)
Categories		
Quartile1	0(ref)	1(ref)
Quartile2	0.97 (0.71, 1.23)	1.83 (1.46, 2.28)
Quartile3	1.48 (1.18, 1.78)	2.66 (2.07, 3.42)
Quartile4	1.96 (1.60, 2.32)	3.37 (2.48, 4.58)
*P* for trend	<0.001	<0.001
Fully adjusted model (model 3)		
Continuous	1.45 (1.11, 1.79)	2.97 (2.13, 4.14)
Categories		
Quartile1	0(ref)	1(ref)
Quartile2	0.74 (0.48, 1.00)	1.55 (1.23, 1.95)
Quartile3	0.97 (0.68, 1.27)	1.89 (1.45, 2.47)
Quartile4	1.18 (0.82, 1.54)	2.05 (1.47, 2.85)
*P* for trend	<0.001	<0.001

Model 1: No covariates were adjusted.

Model 2: Age, gender, and race were adjusted.

Model 3: Age, gender, race, education level, marital status, PIR, smoking status, alcohol drinking status, diabetes status, hypertension status, CVD, and stroke were adjusted.

**Table 3:** Analysis of the threshold effect between METS-VF and PHQ-9 score and depression in overweight or obese patients.

Outcome	PHQ-9 score	Depression
	β(95%CI)	OR (95% CI)
Fitting by standard linear model	1.45 (1.11, 1.79)	2.97 (2.13, 4.14)
*P*-value	<0.0001	<0.0001
Fitting by two-piecewise linear model		
Breakpoint(K)	7.03	6.99
OR1< K	1.41 (1.06, 1.75)	2.82 (2.00, 3.95)
	<0.0001	<0.0001
OR2> K	2.72 (0.40, 5.03)	9.05 (1.81, 45.26)
	0.0214	0.0073
Logarithmic likelihood ratio test P-value	0.277	0.175

The associations were adjusted for gender, age, race, education level, marital status, PIR, smoking status, alcohol drinking status, diabetes status, hypertension status, CVD, and stroke.

### 3.3 Subgroup analyses

**Figure 3:**
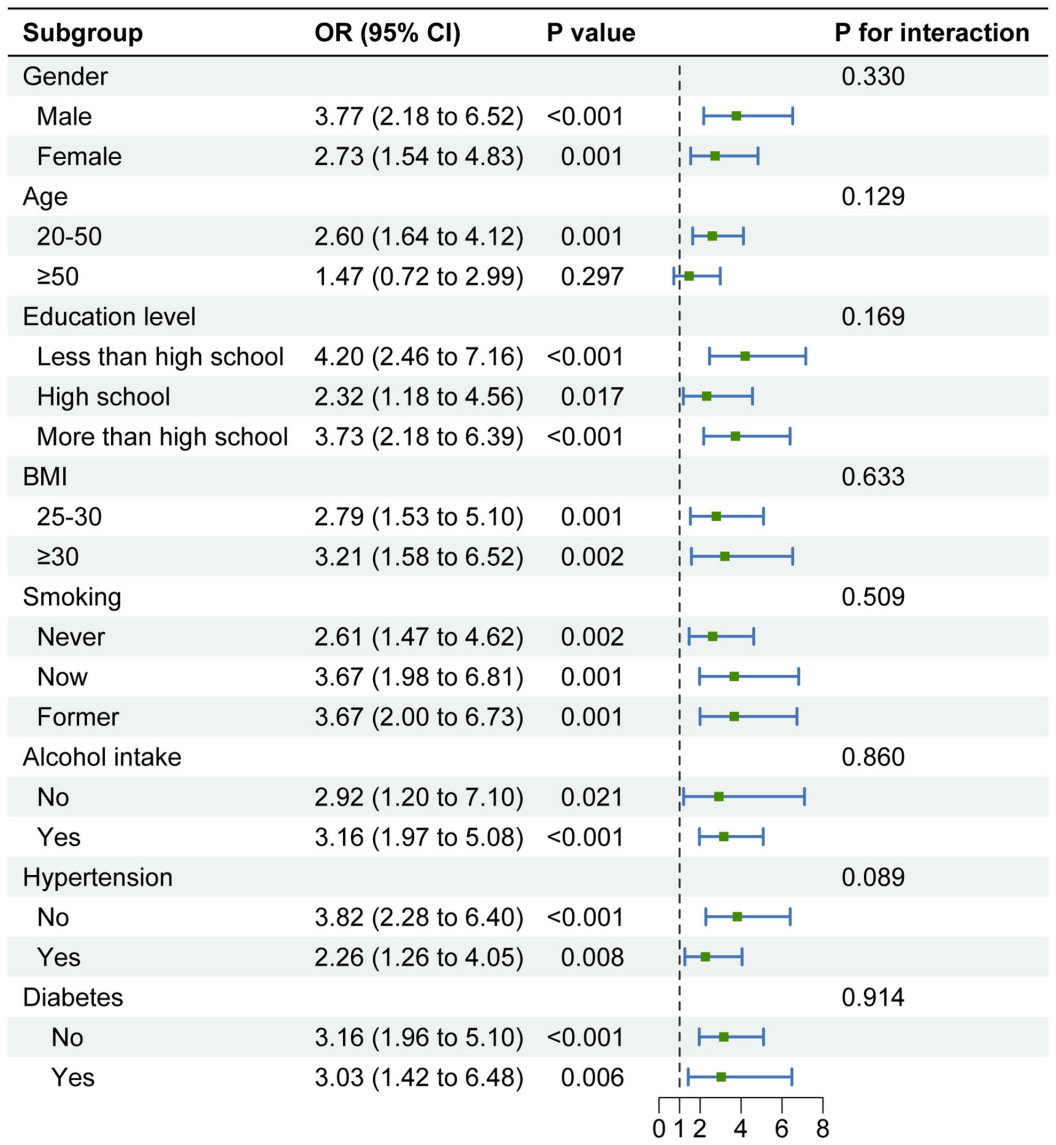
Subgroup analysis of the association between METS-VF and depression in overweight or obese patients. Note 1: The above model adjusted for gender, age, race, education level, marital status, PIR, smoking status, alcohol drinking status, diabetes status, hypertension status, CVD, and stroke. Note 2: In each case, the model is not adjusted for the stratification variable.

Subgroup analyses were conducted to evaluate the consistency of the relationship between METS-VF levels and the prevalence of depression across different populations. The results indicated a positive correlation between METS-VF and depression prevalence in all subgroups, with no significant differences in the relationship across different populations (*P* for interaction > 0.05) (**Figure 3**).

### 3.4 Sensitivity analysis

We extracted NHANES data from 2011-2018 to re-analyze the association between METS-VF and depression in overweight or obese populations as a sensitivity analysis (**Supplementary Figure S1**). The study demonstrated that in the fully adjusted model, METS-VF was positively associated with PHQ-9 scores (β=1.42, 95% CI: 0.84, 1.64). Similarly, METS-VF was positively associated with depression (OR=2.98, 95% CI: 1.98, 4.49) (**Table 4**). Smoothed curve-fitting analyses confirmed that there was no non-linear relationship between METS-VF and depression or PHQ-9 scores (log-likelihood ratio test p-value > 0.05) (**Supplementary Figure S2**) (**Supplementary Table S2**). These findings were consistent with the results of the data analyzed from 2007-2018.

**Table 4:** Multiple regression analysis between METS-VF and depression in overweight or obese patients, 2011-2018.

METS-VF	PHQ-9 score	Depression
	β(95%CI)	OR (95%CI)
Crude model (model 1)		
Continuous	0.60 (0.33, 0.86)	1.74 (1.39, 2.19)
Categories		
Quartile1	0(ref)	1(ref)
Quartile2	0.42 (0.11, 0.72)	1.47 (1.13, 1.92)
Quartile3	0.71 (0.40, 1.01)	1.86 (1.44, 2.40)
Quartile4	0.45 (0.14, 0.75)	1.60 (1.23, 2.08)
*P* for trend	<0.001	<0.001
Minimally adjusted model (model 2)		
Continuous	1.83 (1.43, 2.23)	4.54 (3.08, 6.69)
Categories		
Quartile1	0(ref)	1(ref)
Quartile2	0.70 (0.37, 1.02)	1.73 (1.30, 2.30)
Quartile3	1.28 (0.91, 1.64)	2.59 (1.89, 3.54)
Quartile4	1.61 (1.17, 2.05)	3.12 (2.13, 4.57)
*P* for trend	<0.001	<0.001
Fully adjusted model (model 3)		
Continuous	1.24 (0.84, 1.64)	2.98 (1.98, 4.49)
Categories		
Quartile1	0(ref)	1(ref)
Quartile2	0.52 (0.21, 0.84)	1.52 (1.13, 2.04)
Quartile3	0.82 (0.46, 1.18)	1.90 (1.36, 2.66)
Quartile4	0.96 (0.52, 1.40)	2.08 (1.38, 3.13)
*P* for trend	<0.001	<0.001

Model 1: No covariates were adjusted.

Model 2: Age, gender, and race were adjusted.

Model 3: Age, gender, race, education level, marital status, PIR, smoking status, alcohol drinking status, diabetes status, hypertension status, CVD, and stroke were adjusted.

## 4 Discussion

In populations that are overweight or obese, this research revealed a strong positive correlation between METS-VF and both the likelihood of developing depression and the severity of depression. This implies that higher METS-VF may be an important risk factor for depression. This association was also validated in multiple regression analyses and subgroup analyses and remained significant after adjusting for multiple covariates. These findings suggest that METS-VF may serve as a predictor of depression severity and the risk of developing depression. The sensitivity analyses confirmed the robustness of the results in this study.

In this study, after accounting for potential confounding variables, a strong association was found between elevated levels of the METS-VF index and an increased likelihood of developing depression compared with the low-level group. This finding is consistent with the relationship depicted in **Figure 2**. However, in the subgroup analyses, we observed that this trend may not apply to those over 50 years of age. This situation may offer some new insights, but due to the small sample sizes in the individual strata, it may be influenced by factors such as indicator assessment methods, population characteristics, and false-negative results. Therefore, larger studies with expanded population samples are necessary to account for this relationship in multiple ways. Nevertheless, our findings have implications for understanding the association between visceral fat levels and depression.

Obesity is a serious chronic disease that can be highly detrimental to one’s health (26).

Currently, BMI is commonly used to measure the degree of obesity, and VAI and SAD are also used to measure visceral fat levels (26, 27). However, these metrics have some shortcomings in measuring depression in both men and women (28). Therefore, the Metabolic Score for Visceral Fat (METS-VF) was developed (17, 18). Analysis of research predicting visceral fat using several indices revealed that the METS-VF had the best predictive value for identifying elevated visceral fat (17). This finding confirms previous studies that suggest depression severity and the risk of developing depression are associated with visceral fat distribution in overweight or obese populations.

In studies on the mechanistic aspects of higher levels of visceral adiposity and depression, although the specific mechanisms are not consistent, several possibilities have been suggested. Firstly, Capuron and Dixit believe that inflammation plays a mediating role between obesity and depression, with systemic low-grade inflammation being a key factor in the development of obesity (29, 30), and Margaret suggests that obesity is an important factor that affects the acquired immune system of inflammatory conditions, while adipocytes and infiltrating macrophages distributed in adipose have the ability to secrete inflammatory mediators (31, 32). The inflammatory cytokines generated cause changes in neuroplasticity and brain circuitry, disrupt neurotransmitter metabolism and function, and stimulate the neuroendocrine system (33). Systemic low-grade inflammation has been identified as a strong predictor of depression symptoms in people with metabolic syndrome (34). Secondly, metabolic syndrome plays a role in the development and progression of depression, and it includes obesity. In a study conducted in the UK, the results also showed that metabolic syndrome was associated with an increased risk of depression (35). Thus, there is a possibility that those with high cholesterol have an increased chance of developing depression. Cholesterol affects serotonin levels, which are negatively correlated with the risk of suicide. According to research by Marko, serotonin dysregulation may be linked to both high and low cholesterol levels (36–38). Through several methods, primary reductions in levels of cholesterol might directly lead to decreased brain 5-HT activity. Cholesterol levels may have a considerable impact on the clinical manifestations of depression as well as on the response to psychopharmacological treatment. Individuals with high cholesterol are more likely to experience anxiety problems and exhibit treatment resistance (39). The development of depression is associated with dyslipidemia. A study of lipid levels in depressed postpartum women found that the higher the depression level score, the more abnormal the lipid levels (38). This may be because depression leads to disorders of lipid metabolism, affecting the HPA axis in the body. This, in turn, alters lipid levels and promotes the development of depression (40). There is evidence to show that in people with depression, the axis of HPA may elevate glucocorticoid hormone levels, which may impact insulin’s ability to promote glucose absorption. This permits the body to store fat and blood sugar, which increases metabolic disorders (41). However, research examining the connection between teenage depression and lipid profiles discovered that the degree of dyslipidemia in teenagers with severe depressive disorder was similar to that of teenagers in good health (42). Thus, further investigation is still required for later studies on the connection between lipid levels and the incidence of depression in individuals of all ages. Nevertheless, METS-VF can more accurately represent visceral fat levels and offer a strong foundation for halting the onset and progression of depression.

## 5 Strengths and limitations

The strengths of this study include, first, the feasibility and usefulness of METS-VF as a novel index for assessing visceral fat levels. Second, the large sample size and high representativeness of the study population enhance the robustness of the findings. However, there are several limitations. First, due to the lack of information in the NHANES dataset on whether participants were treated with anti-obesity medications or had undergone bariatric surgery, we were unable to assess the potential impact of these factors on the results. Moreover, we did not analyze risk factors contributing to the development of depression from a microbial mechanism perspective. Additionally, the cross-sectional nature of the analyses limits our ability to infer a causal relationship between METS-VF and depression. Nonetheless, this study provides valuable insights into the relationship between visceral fat levels and depression severity and lays the foundation for future research.

## 6 Conclusion

The present study demonstrated a significant positive association between METS-VF and the likelihood of developing depression in overweight or obese individuals. Future research should adopt a longitudinal design to explore the complex relationship between visceral fat metabolism and depression in greater depth.

## Data Availability

The original contributions presented in the study are included in the article/**Supplementary Material**. Further inquiries can be directed to the corresponding author.
