# Supplementary material for: The association between Metabolic Score for Visceral Fat and depression in overweight or obese individuals: evidence from NHANES
Source: Front Endocrinol (Lausanne). 2024 Sep 26;15:1482003. doi: 10.3389/fendo.2024.1482003 (PMC11464326; doi:10.3389/fendo.2024.1482003)
Supplement: Supplementary file 1 [file DataSheet1.docx]

**The Association Between Metabolic Score for Visceral Fat (METS-VF) and Depression in Overweight or Obese Individuals: Evidence from NHANES**

**Figure S1.** Flow chart of sample selection from the NHANES 2011–2018.

**
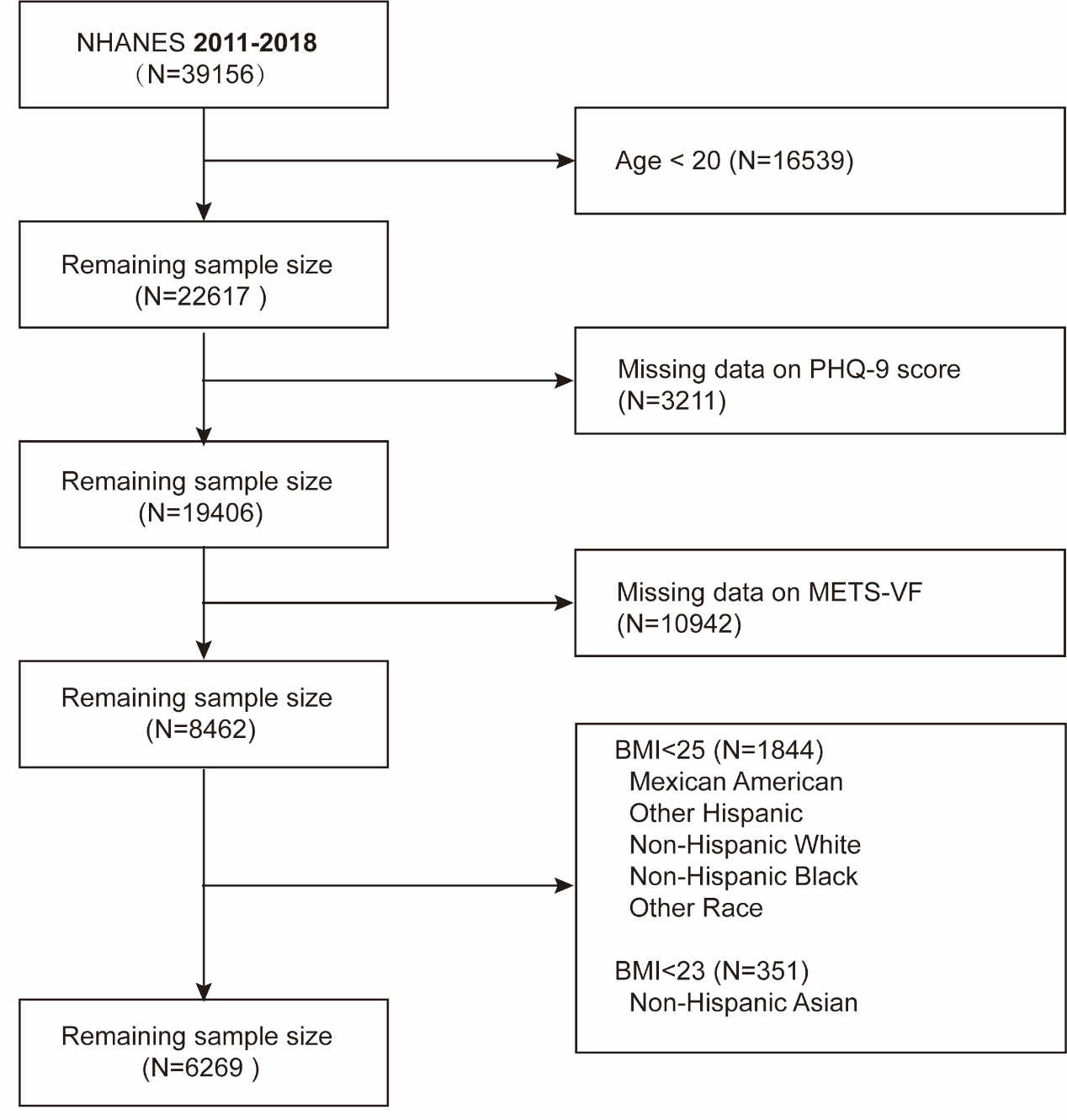
**

**Table S1.** Depression-weighted characteristics of the study population, 2011-2018.

| **Characteristics** | **Total (n = 6269)** | **PHQ-9 <10 (n = 5696)** | **PHQ-9 ≥10 (n = 573)** | **P-value** |
| --- | --- | --- | --- | --- |
| Age(years) | 49.30 (48.64,49.97) | 49.29 (48.57,50.00) | 49.51 (47.92,51.10) | 0.8058 |
| Gender (%) |  |  |  | <0.0001 |
| Male | 51.59 (50.20,52.98) | 53.09 (51.66,54.51) | 34.13 (29.29,39.34) |  |
| Female | 48.41 (47.02,49.80) | 46.91 (45.49,48.34) | 65.87 (60.66,70.71) |  |
| Race (%) |  |  |  | <0.0001 |
| Mexican American | 9.80 (7.76,12.30) | 9.89 (7.82,12.44) | 8.73 (6.09,12.36) |  |
| Other Hispanic | 6.36 (5.15,7.83) | 6.13 (4.94,7.59) | 9.02 (6.18,12.97) |  |
| Non-Hispanic White | 65.67 (61.96,69.20) | 65.99 (62.21,69.58) | 61.92 (54.77,68.59) |  |
| Non-Hispanic Black | 10.53 (8.75,12.62) | 10.36 (8.59,12.46) | 12.44 (9.48,16.16) |  |
| Non-Hispanic Asian | 4.39 (3.61,5.33) | 4.63 (3.81,5.62) | 1.57 (0.94,2.60) |  |
| Other Race | 3.25 (2.55,4.14) | 2.99 (2.32,3.84) | 6.33 (3.92,10.06) |  |
| Education level (%) |  |  |  | <0.0001 |
| Less than high school | 14.64 (13.07,16.36) | 13.88 (12.28,15.64) | 23.48 (19.47,28.02) |  |
| High school | 23.45 (21.69,25.31) | 23.11 (21.28,25.04) | 27.41 (23.05,32.26) |  |
| More than high school | 61.92 (59.33,64.43) | 63.02 (60.30,65.65) | 49.11 (43.91,54.33) |  |
| Marital status (%) |  |  |  | <0.0001 |
| Never married | 15.22 (13.57,17.03) | 14.82 (13.08,16.74) | 19.84 (15.50,25.03) |  |
| Married/Living with partner | 65.93 (63.73,68.07) | 67.60 (65.25,69.86) | 46.55 (40.33,52.88) |  |
| Widowed/divorced/Separated | 18.85 (17.43,20.37) | 17.59 (16.10,19.18) | 33.61 (28.99,38.57) |  |
| PIR (%) |  |  |  | <0.0001 |
| <1.3 | 22.71 (20.68,24.87) | 20.72 (18.88,22.68) | 45.87 (38.99,52.91) |  |
| 1.3 - 3.5 | 36.33 (34.18,38.54) | 36.43 (34.27,38.64) | 35.24 (29.91,40.96) |  |
| ≥3.5 | 40.96 (37.97,44.02) | 42.86 (39.88,45.89) | 18.89 (13.82,25.29) |  |
| BMI (%) |  |  |  | <0.0001 |
| overweight | 45.55 (44.05,47.07) | 46.81 (45.16,48.47) | 30.91 (26.01,36.29) |  |
| obese | 54.45 (52.93,55.95) | 53.19 (51.53,54.84) | 69.09 (63.71,73.99) |  |
| Smoking status (%) |  |  |  | <0.0001 |
| Never | 54.97 (53.03,56.89) | 56.25 (54.18,58.31) | 39.97 (34.80,45.37) |  |
| Now | 16.75 (15.34,18.27) | 15.24 (13.86,16.73) | 34.34 (28.25,40.98) |  |
| Former | 28.28 (26.45,30.19) | 28.50 (26.66,30.42) | 25.70 (20.49,31.70) |  |
| Alcohol intake (%) |  |  |  | 0.0041 |
| No | 14.53 (12.74,16.53) | 14.07 (12.29,16.06) | 19.90 (15.59,25.05) |  |
| Yes | 85.47 (83.47,87.26) | 85.93 (83.94,87.71) | 80.10 (74.95,84.41) |  |
| Hypertension (%) |  |  |  | 0.0004 |
| No | 54.24 (52.23,56.24) | 55.04 (52.91,57.15) | 44.90 (39.68,50.23) |  |
| Yes | 45.76 (43.76,47.77) | 44.96 (42.85,47.09) | 55.10 (49.77,60.32) |  |
| Diabetes (%) |  |  |  | 0.0003 |
| No | 80.54 (79.10,81.90) | 81.13 (79.55,82.62) | 73.61 (69.54,77.32) |  |
| Yes | 19.46 (18.10,20.90) | 18.87 (17.38,20.45) | 26.39 (22.68,30.46) |  |
| Stroke (%) |  |  |  | <0.0001 |
| No | 97.07 (96.52,97.54) | 97.47 (96.80,98.00) | 92.44 (89.16,94.79) |  |
| Yes | 2.93 (2.46,3.48) | 2.53 (2.00,3.20) | 7.56 (5.21,10.84) |  |
| CVD (%) |  |  |  | <0.0001 |
| No | 91.87 (90.97,92.68) | 92.39 (91.38,93.29) | 85.80 (82.02,88.89) |  |
| Yes | 8.13 (7.32,9.03) | 7.61 (6.71,8.62) | 14.20 (11.11,17.98) |  |
| FBG (mg/dl) | 111.16 (109.98,112.33) | 110.59 (109.37,111.81) | 117.74 (113.66,121.82) | 0.0018 |
| HDL-C (mg/dl) | 51.19 (50.60,51.79) | 51.34 (50.73,51.95) | 49.48 (48.27,50.69) | 0.0036 |
| TG (mg/dl) | 133.63 (129.11,138.16) | 132.09 (127.59,136.59) | 151.60 (140.14,163.06) | 0.0008 |
| WHTR | 0.63 (0.63,0.64) | 0.63 (0.63,0.63) | 0.67 (0.66,0.68) | <0.0001 |
| METS-IR | 2.40 (2.39,2.40) | 2.39 (2.38,2.40) | 2.45 (2.43,2.48) | <0.0001 |
| METS-VF | 6.51 (6.49,6.52) | 6.50 (6.49,6.52) | 6.57 (6.54,6.60) | 0.0006 |

For continuous variables: survey-weighted mean (95% CI), with the *P*-value determined by survey-weighted linear regression. For categorical variables: survey-weighted percentage (95% CI), with the P-value determined by survey-weighted Chi-square test.

Abbreviations: PIR, the ratio of income to poverty; BMI, body mass index; HDL-C, high-density lipoprotein cholesterol; TG, triglyceride; WHTR, waist-to-height ratio; FBG, fasting blood glucose; CVD, cardiovascular disease; METS-IR, metabolic score for insulin resistance; METS-VF, metabolic score for visceral fat; PHQ-9, Patient Health Questionnaire-9.

**Table 4.** Multiple regression analysis between METS-VF and depression in overweight or obese patients, 2011-2018.

| **METS-VF** | **PHQ-9 score** |  | **Depression** |
| --- | --- | --- | --- |
|  | **β(95%CI)** |  | **OR (95%CI)** |
| Crude model (model 1) |  |  |  |
| Continuous | 0.60 (0.33, 0.86) |  | 1.74 (1.39, 2.19) |
| Categories |  |  |  |
| Quartile1 | 0(ref) |  | 1(ref) |
| Quartile2 | 0.42 (0.11, 0.72) |  | 1.47 (1.13, 1.92) |
| Quartile3 | 0.71 (0.40, 1.01) |  | 1.86 (1.44, 2.40) |
| Quartile4 | 0.45 (0.14, 0.75) |  | 1.60 (1.23, 2.08) |
| *P* for trend | <0.001 |  | <0.001 |
| Minimally adjusted model (model 2) |  |  |  |
| Continuous | 1.83 (1.43, 2.23) |  | 4.54 (3.08, 6.69) |
| Categories |  |  |  |
| Quartile1 | 0(ref) |  | 1(ref) |
| Quartile2 | 0.70 (0.37, 1.02) |  | 1.73 (1.30, 2.30) |
| Quartile3 | 1.28 (0.91, 1.64) |  | 2.59 (1.89, 3.54) |
| Quartile4 | 1.61 (1.17, 2.05) |  | 3.12 (2.13, 4.57) |
| *P* for trend | <0.001 |  | <0.001 |
| Fully adjusted model (model 3) |  |  |  |
| Continuous | 1.24 (0.84, 1.64) |  | 2.98 (1.98, 4.49) |
| Categories |  |  |  |
| Quartile1 | 0(ref) |  | 1(ref) |
| Quartile2 | 0.52 (0.21, 0.84) |  | 1.52 (1.13, 2.04) |
| Quartile3 | 0.82 (0.46, 1.18) |  | 1.90 (1.36, 2.66) |
| Quartile4 | 0.96 (0.52, 1.40) |  | 2.08 (1.38, 3.13) |
| *P* for trend | <0.001 |  | <0.001 |

Model 1: No covariates were adjusted.

Model 2: Age, gender, and race were adjusted.

Model 3: Age, gender, race, education level, marital status, PIR, smoking status, alcohol drinking status, diabetes status, hypertension status, CVD, and stroke were adjusted.

**Figure S2.** (A) Smooth curve fitting between METS-VF and PHQ-9 Score. (B) Smooth curve fitting between METS-VF and Depression (2011-2018).

**
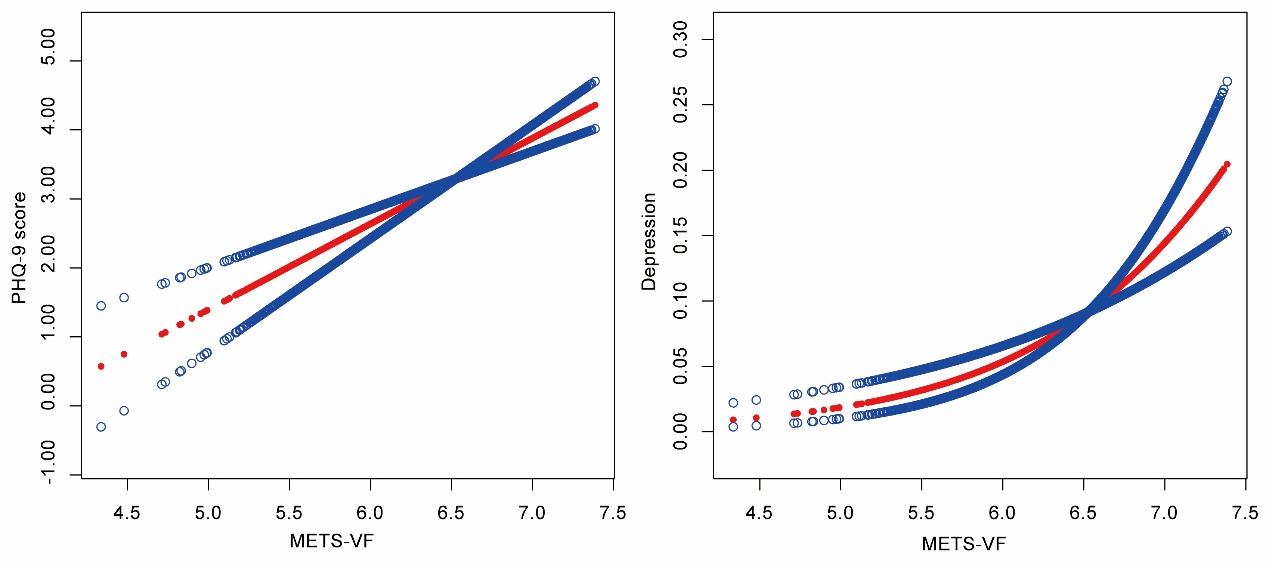
**

The associations were adjusted for gender, age, race, education level, marital status, PIR, smoking status, alcohol drinking status, diabetes status, hypertension status, CVD, and stroke.

**Table S2.** Analysis of the threshold effect between METS-VF and PHQ-9 score and depression in overweight or obese patients, 2011-2018.

| Outcome | PHQ-9 score | Depression |
| --- | --- | --- |
|  | **β**(95% CI) | OR (95% CI) |
| Fitting by standard linear model | 1.24 (0.84, 1.64) | 2.98 (1.98, 4.49) |
| P-value | <0.0001 | <0.0001 |
| Fitting by two-piecewise linear model |  |  |
| Breakpoint(K) | 7.05 | 7.06 |
| OR1< K | 1.15 (0.74, 1.56) | 2.76 (1.82, 4.18) |
|  | <0.0001 | <0.0001 |
| OR2> K | 4.56 (1.45, 7.67) | 28.76 (2.37, 349.38) |
|  | <0.0001 | 0.0084 |
| Logarithmic likelihood ratio test P-value | 0.055 | 0.080 |

The associations were adjusted for gender, age, race, education level, marital status, PIR, smoking status, alcohol drinking status, diabetes status, hypertension status, CVD, and stroke.
